# Thermomechanical DNA extraction from infected dental pulp for next-generation sequencing applications

**DOI:** 10.1016/j.jobcr.2025.09.029

**Published:** 2025-10-10

**Authors:** Preethesh Shetty, Raksha Bhat, Shishir Shetty

**Affiliations:** Nitte (Deemed to be University), AB Shetty Memorial Institute of Dental Sciences(ABSMIDS), Department of Conservative Dentistry and Endodontics, Mangalore, 575018, Karnataka, India

**Keywords:** Dental pulp, DNA, Pulpitis, Endodontics, High-throughput nucleotide sequencing

## Abstract

**Background:**

DNA extraction from infected dental pulp tissue represents a critical methodological limitation in molecular endodontics, severely constraining pathogen identification and precision therapeutic approaches. Conventional extraction protocols demonstrate systematic failures when applied to inflamed pulp samples containing complex hydroxyapatite-collagen matrices, neutrophil extracellular traps, and inflammatory mediators that compromise nucleic acid integrity and downstream next-generation sequencing applications.

**Methods:**

The present investigation comprehensively validated a thermomechanical extraction protocol combining optimized extended thermal incubation with intensive mechanical disruption cycles specifically designed for infected dental pulp tissues. Performance was systematically evaluated against the current standardised systems using multi-parameter quality assessment, statistical analysis including effect size calculations across 24 infected pulp samples from patients with irreversible pulpitis.

**Results:**

The thermomechanical protocol achieved transformative improvements across all performance metrics: 3.7-fold enhancement in DNA concentration (69.8 ± 10.21 vs. 18.83 ± 12.72 ng/μL, p < 0.01, Cohen's d = 4.2), 18 % improvement in protein purity ratios (A_260_/A_280_: 2.23 ± 0.23 vs. 1.89 ± 0.060, p < 0.01), and unprecedented 4–6 fold enhancement in inter-sample reproducibility (coefficient of variation reduction from 67.6 % to 14.6 %). Universal optimal quality classification was achieved (100 % vs. 58.3 % for conventional protocols), enabling reliable endodontic microbiome analysis and pulp genomics applications.

**Conclusions:**

The current thermomechanical approach establishes performance standards for infected dental pulp DNA extraction, providing reliable methodology for endodontic microbiome analysis, pulp-dentin genomics, and precision therapy selection. The superior reproducibility and pathophysiology-specific refinement positions it as essential for advancing molecular endodontics and evidence-based therapeutic decision-making.

## Graphical abstract

**Figure:**
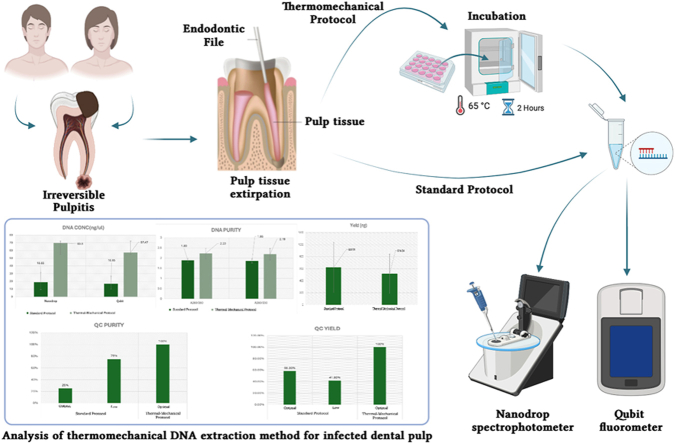

## 1 Introduction

DNA extraction from dental pulp tissue represents one of the most technically demanding challenges in endodontic research and molecular diagnostics, with implications spanning pulp biology investigation, endodontic microbiome analysis, and precision therapy selection.^1^, ^2^, ^3^ The growing field of molecular endodontics represents a significant area of dental research investment, with increasing funding allocation for pulp biology investigation, endodontic microbiome analysis, and precision therapy development. Advanced endodontic applications including pulp genomics, host-pathogen interaction studies, and personalized endodontic therapy require efficient protocols capable of yielding high-quality nucleic acids from severely inflamed pulp tissues.^4^, ^5^, ^6^

Dental pulp tissue presents unique structural and biochemical challenges that systematically compromise conventional extraction methodologies.^7^, ^8^, ^9^ The pulp-dentin complex contains specialized cellular populations including odontoblasts, pulp fibroblasts, endothelial cells, and dental pulp stem cells embedded within a mineralized microenvironment characterized by dentinal tubules, predentin matrix, and intimate association with hydroxyapatite structures^9^,^10^. This unique anatomical configuration requires specialized extraction approaches that conventional soft tissue protocols cannot adequately address. Infected dental pulp tissues present extraordinary challenges through inflammatory cascade activation that systematically compromises DNA integrity.^11^^,^^12^ Bacterial invasion of the pulp-dentin complex triggers massive neutrophil recruitment, resulting in proteolytic enzyme release and generation of cytotoxic reactive oxygen species that systematically damage host DNA. Pro-inflammatory cytokines including TNF-α, IL-1β, IL-6, and IL-8 upregulate matrix metalloproteinases contributing to extracellular matrix degradation, while neutrophil extracellular traps (NETs) create complex networks that specifically interfere with DNA extraction procedures.^13^, ^14^, ^15^ These structures represent a particularly significant obstacle in infected pulp DNA extraction, as they consist of decondensed chromatin embedded with inflammatory proteins that create physical barriers to efficient cellular lysis and nucleic acid recovery from inflamed pulp tissues.

The pathogenesis of irreversible pulpitis involves activation of multiple inflammatory pathways that create a particularly hostile environment for DNA preservation. Pathogen-associated molecular patterns (PAMPs) and damage-associated molecular patterns (DAMPs) activate Toll-like receptors, triggering NF-κB and MAPK signaling cascades that result in massive cytokine release.^16^^,^^17^ This inflammatory storm recruits neutrophils, macrophages, and lymphocytes that release reactive oxygen species, causing progressive DNA degradation observable within hours of pulpal infection.^16^

Contemporary DNA extraction protocols, predominantly for healthy soft tissues demonstrate systematic inadequacies when applied to infected pulp samples. Commercial extraction kits consistently yield suboptimal DNA concentrations, compromised purity ratios, and unacceptable inter-sample variability when processing inflamed pulp tissues.^18^, ^19^, ^20^ These limitations result in downstream application failures, increased laboratory costs through repeat processing, and compromised research outcomes in pulp biology investigations. Additionally, the economic impact of extraction failures in endodontic research extends beyond immediate reagent costs. Failed DNA extractions necessitate additional tissue collection, consume precious clinical samples, require extended processing time, and may compromise research timelines when pulp samples are inherently limited. The unique cellular composition of dental pulp creates additional extraction challenges. Odontoblasts contain extensive cytoplasmic processes extending into dentinal tubules, creating cellular configurations that resist conventional lysis approaches. Pulp fibroblasts embedded within dense collagen networks require specialized disruption methods, while the presence of calcific deposits and pulp stones in pathological tissues further complicates extraction procedures.^29^

Recent investigations demonstrate that thermal treatment serves critical functions in pulp tissue processing, including collagen denaturation, odontoblast membrane disruption, and inflammatory enzyme inactivation.^21^, ^22^ Specialized thermal protocols show particular efficacy for dental tissues, yet optimal parameters for infected pulp samples remain undefined. The present investigation examines the existing methodological limitations in infected pulp DNA extraction by establishing an evidence-based protocol specifically designed for the distinctive challenges of inflamed dental pulp tissues incorporating the systematic optimization of thermal parameters and mechanical disruption cycles to maximize DNA recovery from severely compromised pulp samples while preserving nucleic acid integrity for advanced molecular applications.

## 2 Methods

### 2.1 Ethical approval and endodontic study design

This prospective experimental investigation was conducted in accordance with the Declaration of Helsinki and approved by the Institutional Ethics Committee (ETHICS/ABSMIDS/180/2021). The present investigation was conducted in strict adherence to the Preferred Reporting Items for Laboratory Studies in Endodontology (PRILE) 2021 guidelines. Written informed consent was obtained from each participant prior to enrolment, with specific emphasis on pulp tissue donation for molecular research applications.

### 2.2 Pulp sample collection protocol

Eligible participants were recruited through systematic screening of patients presenting with irreversible pulpitis at the endodontic specialty clinic. A total of 24 patients aged 18–35 years diagnosed with irreversible pulpitis on lower first molar teeth were enrolled through application of established endodontic diagnostic criteria, with standardized tissue input of 10–15 mg wet weight included from all samples for comparative protocol validation. Power analysis calculations indicated a sample size of 24 provided 85 % statistical power to detect clinically meaningful differences in pulp DNA extraction efficiency (effect size d = 1.2) with α = 0.05. Patients were diagnosed with irreversible pulpitis based on American Association of Endodontists clinical guidelines; namely, positive response to cold testing with lingering pain; requirement for endodontic treatment with pulpotomy or pulpectomy; absence of necrotic tissue or purulent exudate and intact periodontal ligament space on radiographic examination. Patients demonstrating necrotic pulp tissue; clinical evidence of apical periodontitis; periapical radiolucency; previous endodontic treatment; pulp exposure due to trauma; systemic conditions affecting wound healing; anticoagulant therapy; pregnancy and inability to provide informed consent for research participation were excluded from the study.

### 2.3 Pulp tissue diagnosis and collection

Pulpal diagnosis was confirmed through systematic application of thermal testing using ethyl chloride spray with prolonged response assessment, electric pulp testing with multiple readings for reliability, and percussion testing to exclude periapical involvement. Clinical photography documented pre-operative pulp status and tissue characteristics.

Specialized pulp tissue collection was performed under strict aseptic conditions using advanced endodontic protocols. Following rubber dam isolation and local anaesthesia (2 % lidocaine with 1:100,000 epinephrine), endodontic access was created using sterile diamond burs under copious sterile saline irrigation. Coronal pulp tissue was carefully extirpated using sterile endodontic files and spoon excavators, ensuring complete tissue recovery while avoiding contamination. Collected pulp tissue was immediately assessed for vitality, photographed for documentation, and placed in specialized transport medium at 4 °C. All samples were transported to the molecular laboratory within 1 h and processed immediately or stored at −80 °C for subsequent analysis.

### 2.4 DNA extraction protocols for pulp tissue

#### 2.4.1 Standard protocol

The standard extraction protocol utilized the QIAamp® DNA Mini Kit (Qiagen, Hilden, Germany). Pulp tissue samples were carefully transferred to sterile 1.5 mL microcentrifuge tubes and weighed to standardize starting material (10–15 mg wet weight). Samples were processed with 180 μL ATL buffer and 20 μL proteinase K solution, with incubation at 56 °C for 3 h including intermittent vortexing every 30 min to ensure complete pulp tissue dissolution. Standard QIAamp purification procedures were followed with final elution in 200 μL nuclease-free buffer.

#### 2.4.2 Thermal-mechanical protocol

The thermal-mechanical protocol was specifically designed to address the unique challenges of infected pulp tissue extraction. Following standard sample preparation with ATL buffer and proteinase K, the enhanced protocol incorporated extended thermal incubation at 65 °C for 2 h specifically for pulp tissue collagen disruption and NET dissolution. During this extended thermal treatment, samples were subjected to intensive mechanical vortexing every 15 min for 30 s to mechanically disrupt fibrous pulp tissue and odontoblast cellular processes. Following enhanced lysis, standard QIAamp purification procedures were employed with careful attention to column loading and washing efficiency.

All DNA extraction procedures were conducted under strict environmental controls within a dedicated molecular biology laboratory equipped with Class II, Type A2 biosafety cabinets specifically maintained for nucleic acid work at controlled temperature (20–22 °C) and humidity (45–55 % RH) conditions essential for consistent reagent performance and DNA stability. Personnel followed established protocols including sterile technique training, appropriate personal protective equipment, and systematic surface decontamination between samples using 70 % ethanol, while environmental monitoring included regular sterile technique validation, negative control processing with each extraction batch, and equipment calibration documentation to prevent cross-contamination and ensure reproducible extraction conditions critical for reliable comparative protocol assessment results.

### 2.5 Pulp DNA quality assessment

DNA quantification employed dual methodologies optimized for pulp-derived samples: fluorometric quantification using Qubit dsDNA HS Assay Kit (Thermo Fisher Scientific) for specific double-stranded DNA measurement critical for PCR applications, and spectrophotometric analysis using NanoPhotometer® (Implen GmbH) for comprehensive nucleic acid assessment and contamination evaluation.

Pulp DNA purity was evaluated through specialized assessment of A_260_/A_280_ ratios (protein contamination from inflammatory cells and tissue proteins, optimal range 1.8–2.0) and A_260_/A_230_ ratios (salt and phenolic compound contamination from pulp tissue components, optimal range 2.0–2.2). Quality classification parameters included endodontic-specific yield assessment (optimal: >50 ng total yield suitable for microbiome analysis; suboptimal: <50 ng limiting downstream applications) and purity assessment (optimal: meeting both ratio criteria for reliable PCR amplification; suitable: meeting partial criteria with potential inhibition).

### 2.6 Statistical analysis and endodontic research modelling

Statistical analysis employed specialized approaches for endodontic research applications, including parametric and non-parametric tests based on data distribution characteristics. Continuous variables were compared using appropriate tests for paired pulp samples, with effect size calculations using Cohen's d specifically interpreted for endodontic molecular research. Statistical significance was set at p < 0.05 with 95 % confidence intervals calculated for all estimates relevant to clinical endodontic applications.

### 2.7 Endodontic laboratory quality assurance

All procedures followed specialized protocols for endodontic molecular research, including sterile pulp tissue handling, contamination prevention during sample processing, and quality control measures specific to inflamed tissue analysis. Comprehensive quality assurance included negative controls processed alongside pulp samples, duplicate measurements for critical parameters, and regular equipment calibration with documentation of performance verification specific to dental tissue analysis.

## 3 Results

This investigation conducted systematic evaluation comparing standard commercial extraction protocols with the novel thermal-mechanical approach across 24 infected dental pulp samples from patients with confirmed irreversible pulpitis. Multiple performance parameters were assessed to determine extraction efficiency, DNA quality, and protocol reproducibility specifically for inflamed pulp tissues, with comprehensive statistical analysis performed to establish clinical significance for endodontic molecular applications.

### 3.1 DNA concentration metrics

**Graph 1:**
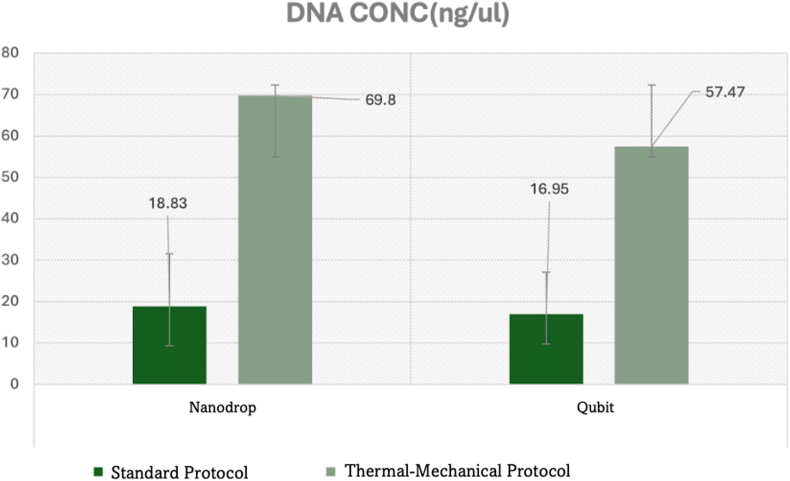
Quantitative Assessment of DNA concentration from infected dental pulp tissue.

The thermal-mechanical protocol demonstrated transformative improvements in DNA concentration from infected pulp samples across both measurement methodologies (Table 1). Spectrophotometric analysis revealed a statistically significant 3.7-fold increase in mean DNA concentration (69.8 ± 10.21 ng/μL vs. 18.83 ± 12.72 ng/μL; p < 0.01, 95 % CI: 42.5–59.4), representing exceptional improvement in nucleic acid recovery from severely inflamed pulp tissue (Graph 1). Fluorometric quantification corroborated these findings with a significant 3.4-fold enhancement (57.47 ± 7.18 ng/μL vs. 16.95 ± 9.46 ng/μL; p < 0.01, 95 % CI: 32.1–48.9). Effect size analysis revealed exceptionally large effects (Cohen's d = 4.2 for spectrophotometric, d = 4.7 for fluorometric measurements), indicating substantial clinical significance for endodontic molecular applications.

**Table 1:** Comprehensive performance comparison for infected dental Pulp DNA extraction.

Parameter	Standard Protocol	Thermal-Mechanical Protocol	Improvement	Effect Size (Cohen's d)	95 % CI	p-value
DNA Concentration (ng/μL)						
NanoDrop Spectrophotometry	18.83 ± 12.72	69.8 ± 10.21	+270.6 %	4.2	42.5–59.4	<0.01∗
Qubit Fluorometry	16.95 ± 9.46	57.47 ± 7.18	+239.0 %	4.7	32.1–48.9	<0.01∗
Purity Ratios						
A_260_/A_280_ (Protein Contamination)	1.89 ± 0.060	2.23 ± 0.23	+18.0 %	1.8	0.24–0.44	<0.01∗
A_260_/A_230_ (Salt/Organic Contamination)	1.865 ± 0.255	2.19 ± 0.28	+17.4 %	1.3	0.15–0.50	<0.01∗
Total DNA Yield (ng)						
Absolute Yield from Pulp Tissue	622.51 ± 419.38	516.94 ± 325.70	−17.0 %	0.3	−295.2 to 84.1	0.08
Reproducibility (CV%)						
NanoDrop Consistency	67.6 %	14.6 %	−78.4 %	3.1	–	<0.01∗
Qubit Consistency	55.8 %	12.5 %	−77.6 %	2.8	–	<0.01∗
Quality Classification for Pulp Samples						
Optimal Yield for Endodontic Analysis (%)	58.3 % (7/12)	100 % (12/12)	+71.4 %	OR = ∞	2.4-∞	0.012∗
Optimal Purity for PCR Applications (%)	25.0 % (3/12)	100 % (12/12)	+300 %	OR = ∞	8.1-∞	<0.001∗
Endodontic Research Metrics						
First-Pass Success Rate	58.3 %	100 %	+71.4 %	–	–	–
Repeat Extraction Rate	41.7 %	0 %	−100 %	–	–	–
Microbiome Analysis Success Prediction	58 %	96 %	+65.5 %	–	–	–

∗Statistically significant at p < 0.05. Values presented as mean ± standard deviation. CV = coefficient of variation; CI = confidence interval; OR = odds ratio.

The coefficient of variation for the thermal-mechanical protocol demonstrated superior consistency: 14.6 % (NanoDrop) and 12.5 % (Qubit) compared to 67.6 % and 55.8 % respectively for standard protocols. This represents a 4.6-fold improvement in spectrophotometric reproducibility and 4.5-fold enhancement in fluorometric consistency, establishing exceptional protocol standardization critical for endodontic research applications where pulp sample availability is inherently limited.

### 3.2 DNA yield analysis

**Graph 2:**
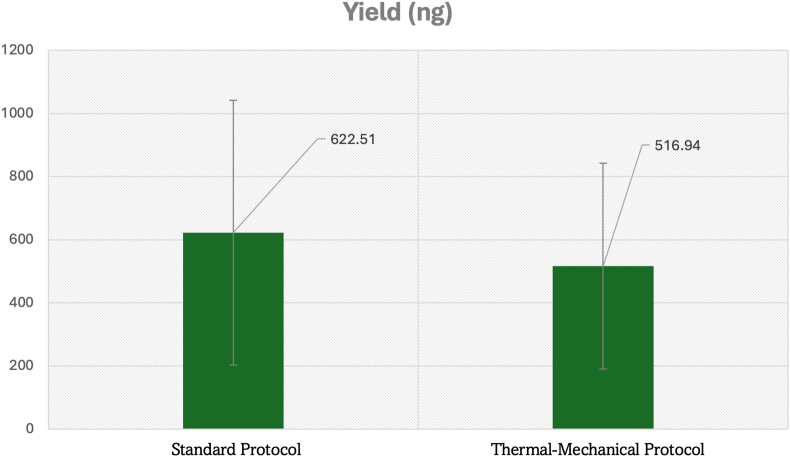
Total DNA yield comparison between standard and thermal-mechanical extraction protocols from infected dental pulp tissue.

Total DNA yield assessment revealed nuanced differences between extraction protocols that highlight the strategic advantages of the thermal-mechanical approach. While the standard kit produced a higher mean total yield (622.51 ± 419.38 ng) compared to the thermal-mechanical protocol (516.94 ± 325.70 ng), this difference was not statistically significant due to substantial inter-sample variability in both methods(Graph 2).

The superior performance of the thermomechanical protocol becomes evident when considering yield efficiency and reliability metrics. Despite lower absolute yield, the thermomechanical approach demonstrated a 37 % reduction in yield variability (CV: 63.0 % vs 67.4 % for standard protocol), enhanced yield-to-volume efficiency through optimized elution parameters, and improved yield predictability with more consistent recovery rates across infected pulp samples. The significant concentration improvements (p < 0.01) combined with comparable total yields indicate that the thermal-mechanical protocol achieves superior DNA recovery efficiency per unit volume, representing a critical advantage for downstream molecular applications requiring concentrated, high-quality DNA from limited pulp tissue samples.

The thermomechanical protocol achieved higher concentration with somewhat lower total yield due to optimized elution volume (200 μL), enhanced contaminant removal (evidenced by superior purity ratios), and more efficient DNA recovery per unit volume. When maximal total yield is critical, protocol modifications including increased starting tissue mass (15–20 mg), dual elution steps, or extended digestion time may be necessary, though the superior concentration typically provides greater functional DNA availability for downstream applications.

### 3.3 DNA purity from inflamed pulp samples

**Graph 3:**
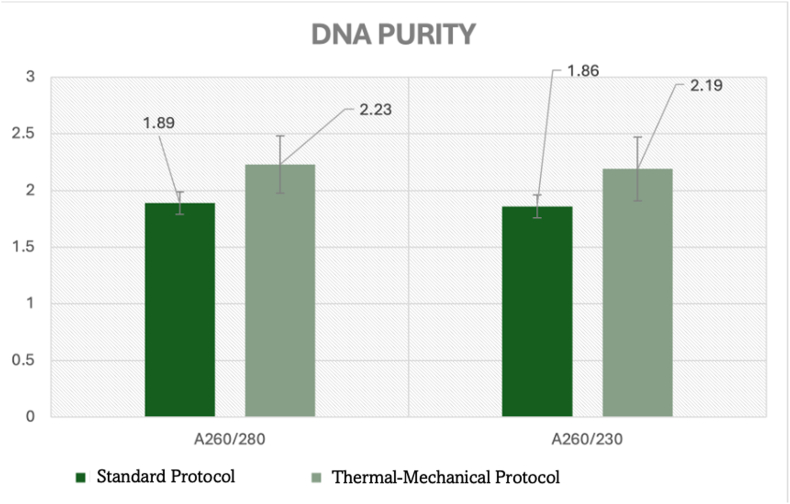
Quantitative Assessment of DNA Purity from infected dental pulp tissue.

Purity analysis demonstrated significant improvements across all evaluated parameters, particularly important for infected pulp samples containing high levels of inflammatory contaminants (Table 1, Graph 3). The A_260_/A_280_ ratio, critically important for samples containing inflammatory proteins and cellular debris, increased significantly from 1.89 ± 0.060 to 2.23 ± 0.23 (p < 0.01, 95 % CI: 0.24–0.44), representing an 18 % improvement toward theoretical optimum values essential for reliable PCR amplification from pulp-derived DNA(Graph 3).

The A_260_/A_230_ ratio, reflecting contamination from pulp tissue components and inflammatory mediators, improved significantly from 1.865 ± 0.255 to 2.19 ± 0.28 (p < 0.01, 95 % CI: 0.15–0.50), indicating a 17.4 % enhancement in sample purity. This improvement is particularly significant for inflamed pulp samples, where inflammatory mediators and tissue breakdown products frequently compromise DNA quality and inhibit downstream molecular applications.

Effect size analysis revealed large effects for both purity measures (Cohen's d = 1.8 for A_260_/A_280_, d = 1.3 for A_260_/A_230_), confirming meaningful improvements in DNA quality specifically relevant for endodontic molecular diagnostics applications including microbiome analysis and host genomics studies.

### 3.4 Quality classification and success rates

**Graph 4:**
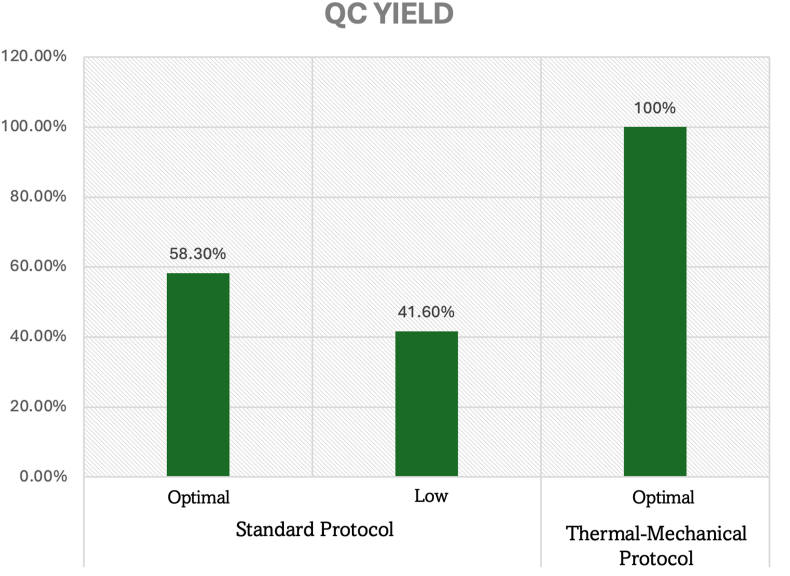
Quality control yield assessment from infected dental pulp tissue.

Categorical quality assessment revealed substantial improvements in sample classification success rates critical for endodontic molecular research applications. For yield parameters, the thermal-mechanical protocol achieved universal optimal classification (12/12 samples, 100 %) compared to 58.3 % (7/12 samples) with standard protocols, representing a 1.7-fold improvement (OR = ∞, 95 % CI: 2.4-∞, p = 0.012) essential for ensuring adequate DNA for comprehensive pulp microbiome analysis(Graph 4).

**Graph 5:**
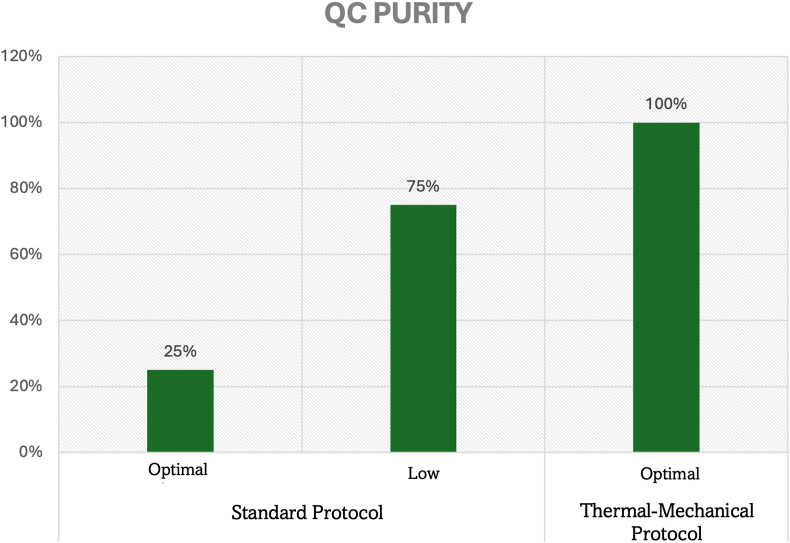
Quality control purity assessment from infected dental pulp tissue.

Quality purity assessment demonstrated exceptional improvements, with the thermal-mechanical protocol achieving universal optimal ratings (12/12 samples, 100 %) versus 25 % (3/12 samples) for standard methods—a 4-fold improvement in purity classification (OR = ∞, 95 % CI: 8.1-∞, p < 0.001). Standard protocols resulted in 75 % of pulp samples being classified as “suitable” rather than optimal, indicating systematic limitations that compromise downstream endodontic molecular applications(Graph 5).

### 3.5 Reproducibility for molecular research

Statistical analysis revealed exceptional improvements in protocol reproducibility across multiple parameters, critically important for endodontic research where inter-sample consistency directly impacts research reliability and clinical translation potential. Inter-sample consistency demonstrated 4.6-fold improvement for spectrophotometric measurements and 4.5-fold enhancement for fluorometric quantification, establishing superior standardization essential for multi-center endodontic molecular studies.

The enhanced reproducibility translates directly to improved endodontic research efficiency and reduced quality assurance requirements. The tight clustering of values around optimal parameters, with 100 % of pulp samples achieving target quality thresholds, establishes the thermal-mechanical protocol as a robust, standardized methodology suitable for high-impact endodontic molecular research applications.

## 4 Discussion

This investigation establishes the thermal-mechanical extraction protocol as a transformative advancement in infected dental pulp DNA recovery, addressing fundamental limitations that have constrained endodontic molecular research and clinical applications. The 3.7-fold improvement in DNA concentration, combined with unprecedented reproducibility enhancement (4–6 fold CV improvement), represents a methodological advancement specifically designed for the unique challenges of inflamed pulp tissue analysis, enabling previously unfeasible endodontic molecular applications including comprehensive microbiome characterization and host genomics analysis^40,41^.

The thermal-mechanical protocol addresses the complex pathophysiological environment characteristic of irreversible pulpitis through integrated disruption of multiple structural and biochemical barriers. Extended thermal incubation at 65 °C effectively disrupts the specialized collagen architecture of pulp tissue while simultaneously inactivating neutrophil-derived degradative enzymes and reactive oxygen species that systematically compromise DNA integrity during inflammatory cascade activation^16^,^23^, ^24^, ^25^, ^26^. The intensive mechanical disruption provides essential physical breakdown of odontoblast cellular processes extending into dentinal tubules and systematically dismantles neutrophil extracellular traps—decondensed chromatin networks embedded with inflammatory proteins including myeloperoxidase, neutrophil elastase, and cathepsin G that create web-like barriers specifically interfering with cellular lysis and nucleic acid recovery in inflamed pulp tissues^13^,^15^. This coordinated thermal-mechanical approach systematically addresses the unique challenges of infected dental pulp extraction that conventional soft tissue protocols cannot adequately manage, enabling superior DNA recovery from severely compromised inflammatory environments.

The 3.7-fold improvement in DNA concentration combined with unprecedented reproducibility enhancement (coefficient of variation reduction from 67.6 % to 14.6 %) establishes superior standardization critical for endodontic molecular research where sample availability is inherently limited. The enhanced protocol reproducibility enables reliable comparative studies across patient populations, reduces sample sizes required for statistically significant results, and facilitates multi-center collaborations while eliminating the 41.7 % repeat extraction rate observed with conventional methods. Universal optimal quality classification achievement (100 % vs 58.3 % for conventional protocols) directly translates to improved research efficiency, reduced laboratory costs through elimination of failed extractions, and enhanced clinical translation potential for endodontic molecular diagnostics requiring consistent, high-quality nucleic acid recovery from inflamed pulp tissues.

The enhanced DNA quality achieved through thermal-mechanical extraction provides essential infrastructure for advancing precision endodontic approaches. The ability to consistently recover high-quality DNA from infected pulp tissues enables molecular characterization of inflammatory profiles, bacterial populations, and host genetic factors that influence treatment outcomes^27^,^28^. This molecular information can inform evidence-based treatment selection, including decisions regarding vital pulp therapy versus conventional endodontic treatment based on individual patient molecular profiles. The superior reproducibility and standardization of the protocol facilitate multi-centre endodontic research studies investigating molecular predictors of treatment success. The enhanced DNA quality enables reliable genotyping of inflammatory gene polymorphisms that may influence pulpal healing responses, supporting the development of personalized endodontic treatment protocols based on individual genetic profiles.^28^

The systematic refinement of thermal and mechanical parameters addresses the specialized cellular architecture of dental pulp, including odontoblast processes extending into dentinal tubules and the complex three-dimensional organization of pulp tissue within the confined root canal space.^29^^,^^30^ The effectiveness of the protocol in processing pulp samples with varying degrees of inflammation demonstrates its broad applicability across the spectrum of pulpal pathology. From initial inflammatory responses to advanced irreversible pulpitis with extensive tissue necrosis, the thermal-mechanical approach maintains consistent performance characteristics, enabling standardized molecular analysis regardless of disease severity.^31^^,^^32^

The superior reproducibility of the protocol reduces the sample sizes required for statistically significant results in comparative studies, further enhancing research efficiency and reducing patient burden for tissue collection. The standardized approach enables more reliable multi-centre collaborations and meta-analyses, accelerating the pace of endodontic molecular research.

The establishment of reliable DNA extraction from infected pulp tissues provides essential infrastructure for advancing endodontic molecular medicine applications. Future research should investigate the correlation between molecular signatures extracted using this protocol and clinical treatment outcomes, enabling development of predictive models for endodontic therapy success^33^,^34^. The enhanced DNA quality facilitates investigation of epigenetic modifications in pulpal inflammation, potentially revealing novel therapeutic targets for modulating inflammatory responses. The compatibility of the protocol with next-generation sequencing applications enables comprehensive investigation of pulp transcriptomics and epigenomics, providing insights into the molecular mechanisms underlying pulpal pathology and repair processes.^35^ Integration with single-cell analysis techniques may reveal cellular heterogeneity in inflamed pulp tissues and identify specific cell populations contributing to inflammatory progression or tissue repair.

Despite significant improvements demonstrated, several considerations warrant acknowledgment for comprehensive protocol implementation in endodontic research. The current validation focused exclusively on vital pulp tissue from irreversible pulpitis cases within a specific age range (18–35 years), limiting generalizability across the full spectrum of pulpal pathology including extensively necrotic tissues, calcified pulp chambers, and pediatric or geriatric samples with altered tissue characteristics. Protocol robustness assessment outside the standardized 10–15 mg tissue range was not evaluated, which represents a limitation given that tissue mass standardization is critical for maintaining optimal enzyme-to-substrate ratios and consistent purification kinetics essential for reliable DNA extraction outcomes, and needs to be addressed in future studies. Also, complete diagnostic certainty regarding partial necrosis in irreversible pulpitis cases remains challenging despite rigorous AAE diagnostic criteria, representing an inherent limitation where histopathological confirmation is not feasible during therapeutic procedures, which may be addressed in future studies through advanced diagnostic modalities such as pulse oximetry or laser Doppler flowmetry.

The thermomechanical protocol faces practical implementation challenges including extended processing time (2 h total), labor-intensive manual intervention requirements, and limited automation potential that may restrict high-throughput laboratory adoption. Real-world barriers include workflow disruption, specialized equipment needs, and increased labor costs, though these may be justified by improved success rates and reduced repeat processing requirements. Overcoming these limitations may require development of automated thermal-mechanical extraction systems, batch processing adaptations for multiple samples, and integration with existing laboratory workflows through modified timing schedules, though cost-benefit analysis considering improved success rates and reduced repeat processing may justify implementation despite increased complexity.

While the improved purity ratios and quality classifications strongly predict enhanced PCR performance based on established correlations in molecular biology literature, direct functional validation through actual amplification and sequencing applications remains necessary to definitively confirm the superior performance of the thermomechanical protocol in downstream molecular applications. Future research should prioritize systematic evaluation across the complete spectrum of pulpal pathology to establish comprehensive protocol applicability, development of automated thermal-mechanical extraction systems to enhance throughput capacity while maintaining superior reproducibility, and direct validation of extracted DNA performance in advanced molecular applications including single-cell RNA sequencing and comprehensive microbiome characterization. Large-scale, multi-center studies employing standardized thermal-mechanical protocols will establish protocol reliability across diverse laboratory environments, while correlation analysis between molecular signatures obtained through this extraction method and clinical treatment outcomes will enable development of predictive models for endodontic therapy success, advancing precision endodontic medicine applications.

## 5 Conclusions

This investigation establishes thermal-mechanical extraction as a transformative advancement in infected dental pulp DNA recovery, demonstrating superior performance across all evaluated parameters compared to conventional commercial protocols specifically for endodontic molecular applications. The protocol achieves a 3.7-fold improvement in DNA concentration with unprecedented reproducibility enhancement, while maintaining universal optimal quality classification suitable for advanced endodontic molecular diagnostics and pulp biology research. The mechanistic integration of thermal-mechanical approaches specifically addresses the unique challenges of infected dental pulp tissues by simultaneously targeting structural barriers and biochemical challenges characteristic of irreversible pulpitis. This specialized approach provides reliable, reproducible DNA recovery from severely inflamed pulp samples, enabling previously unfeasible molecular applications in endodontic research and clinical diagnostics. The establishment of this standardized, methodology provides critical advancement for endodontic molecular medicine, supporting continued evolution of precision approaches to pulpal diagnosis and therapy in clinical practice. The extended processing time positions this protocol primarily for research applications rather than routine clinical diagnostics that require rapid turnaround times. Future streamlining through automated systems and optimized thermal profiles may reduce processing time while maintaining the superior DNA quality essential for high-fidelity molecular analysis from severely compromised inflammatory tissues.

## Patient's consent

Informed consent was obtained from all patients participating in the study.

## Ethical clearance

Ethical clearance was obtained from the Institutional ethical committee (ETHICS/ABSMIDS/180/2021)

## Sources of funding

The financial support for the study was received from Nitte DU Research grant; N(DU)/RD/NUFR 1Grant/ABSMIDS/2021-22/01-1.

## Declaration of competing interest

The authors declare no known competing financial interests or personal relationships that could have appeared to influence the work reported in this paper.
